# The mediating role of white matter hyperintensity in the association between malnutrition and cognitive impairment in patients with cerebral small vessel disease

**DOI:** 10.3389/fneur.2026.1841396

**Published:** 2026-07-27

**Authors:** Weigang Luo, Guisong Zhang, Mengying Lu, Wei Bu, Ling Yang, Yimeng Yang, Huiling Ren

**Affiliations:** 1 Department of Neurology, Hebei Medical University Third Hospital, Shijiazhuang, Hebei, China; 2 Hebei Key Laboratory of Neurodegenerative Disease Mechanism, Shijiazhuang, Hebei, China; 3 Hebei Medical University, Shijiazhuang, Hebei, China; 4 Department of Neurosurgery, Hebei Medical University Third Hospital, Shijiazhuang, Hebei, China

**Keywords:** cognitive impairment, Geriatric Nutritional Risk Index, malnutrition, mediation analysis, white matter hyperintensity

## Abstract

**Background:**

Malnutrition and white matter hyperintensity (WMH) are both considered risk factors for cognitive impairment in patients with cerebral small vessel disease (CSVD). However, the associations among nutritional status, WMH burden, and cognitive function remain unclear. This study aimed to investigate the mediating role of WMH volume in malnutrition and cognitive impairment in CSVD patients.

**Methods:**

We conducted a retrospective cross-sectional study of 383 patients with CSVD. Nutritional status and cognitive function were assessed via the Geriatric Nutritional Risk Index (GNRI) and the Montreal Cognitive Assessment (MoCA), respectively. WMH volumes were quantified using VB-Net automatic segmentation. WMH volumes were natural log-transformed using ln(volume + 1) before linear regression and mediation analyses. The associations among GNRI, WMH volume, and MoCA scores were examined using Spearman correlation, multivariable linear regression, and mediation analyses.

**Results:**

Patients with cognitive impairment had significantly lower GNRI scores compared to those with normal cognition. Patients with cognitive impairment presented with significantly larger volumes of total WMH, deep WMH (DWMH), periventricular WMH (PVWMH), and juxtacortical WMH (JCWMH) compared to patients with normal cognition. Spearman correlation analysis indicated significant correlations between GNRI scores, WMH volumes, and MoCA scores. Multivariable regression analysis showed that higher GNRI scores were associated with higher MoCA scores, whereas greater log-transformed WMH volumes were associated with lower MoCA scores. Mediation analysis showed that log-transformed total WMH significantly mediated the association between GNRI and MoCA scores, accounting for 70.7% of the relative effect, with an indirect effect of 0.1490 and a 95% bootstrap CI of 0.1007–0.2054. Among WMH subtypes, PVWMH showed the largest relative mediation effect, accounting for 72.6%, followed by DWMH and JCWMH, which accounted for 49.1 and 47.5%, respectively.

**Conclusion:**

This study indicates that nutritional status and WMH are both closely associated with cognitive function in patients with CSVD. WMH mediated the association between malnutrition and cognitive impairment in the mediation model. These findings highlight WMH as a potential link between nutritional status and cognitive function, providing a clinically relevant perspective for understanding the association between malnutrition and cognitive impairment.

## 1 Introduction

Cerebral small vessel disease (CSVD) is a primary cause of vascular cognitive impairment (1). White matter hyperintensity (WMH), as a neuroimaging marker of CSVD, is closely associated with cognitive impairment (2). CSVD-related cognitive impairment severely impacts patients’ daily living functions and quality of life. It often coexists with neurodegenerative diseases, including Alzheimer’s disease, further complicating clinical diagnosis and treatment (1). However, effective disease-modifying therapies specifically for CSVD are currently lacking, and clinical management primarily relies on controlling vascular risk factors (3). Therefore, further research into the pathophysiological processes associated with CSVD-related cognitive impairment, along with the exploration of imaging markers and biomarkers for early identification, risk stratification, and evaluation of interventional efficacy, is of significant clinical necessity and urgency (1, 4).

Malnutrition is closely related to cognitive impairment. Studies have shown that older adults with adequate nutritional intake exhibit superior cognitive abilities compared to those who are malnourished (5–7). The Geriatric Nutritional Risk Index (GNRI) is an objective indicator that reflects a patient’s nutritional status and is superior to assessments using BMI or albumin alone (8). Two studies, one on older adults in the United States and the other on older adults in China, have shown that lower GNRI is associated with more severe cognitive impairment, affecting multiple cognitive domains (9, 10). Furthermore, interventions aimed at improving nutritional status are associated with better cognitive outcomes, although the factors underlying these associations remain to be fully elucidated (11, 12).

Current research has demonstrated the significance of malnutrition in cognitive impairment, but the role of WMH in mediating this association remains to be fully explored. WMH has emerged as a crucial neuroimaging marker for vascular cognitive impairment, suggesting underlying CSVD and is associated with cognitive decline (13, 14). The formation of WMH is primarily driven by vascular mechanisms, centering on hemodynamic disturbances and the cumulative damage from vascular risk factors (15, 16). Studies indicate that increased WMH volume is an independent risk factor for cognitive impairment (17). Furthermore, research suggests that, compared to stable WMH, the regression of WMH is associated with preserved overall cognitive function and improved executive function, while the progression of WMH is linked to a decline in overall cognitive performance (13). A study investigating the association between malnutrition and imaging biomarkers of CSVD has shown that malnutrition is associated with increased WMH burden (18). However, to date, the role of WMH as a mediator in the association between malnutrition and cognitive impairment has not been sufficiently investigated. Therefore, this study employed advanced imaging technology to quantitatively assess WMH volume, aiming to explore the associations among CSVD-related cognitive impairment, malnutrition, and WMH, and to analyze the mediating role of WMH volume in the association between malnutrition and cognitive impairment.

## 2 Materials and methods

### 2.1 Study population

This retrospective cross-sectional study included patients in the Department of Neurology, Hebei Medical University Third Hospital, from January 2021 to July 2025. The inclusion criteria were: (1) age ≥18 years; (2) evidence of at least one CSVD imaging marker: white matter hyperintensities, cerebral microbleeds, vascular-origin lacunes, or enlarged perivascular spaces (19); (3) participants completed the Montreal Cognitive Assessment (MoCA). The exclusion criteria were: (1) intracranial or extracranial large artery stenosis ≥50% as shown by MRA; (2) severe hepatic or renal dysfunction; (3) patients with other specific conditions that may affect cognitive function assessment, including anxiety, depression, brain injury, intracranial space-occupying lesions, carbon monoxide poisoning, etc.; (4) patients with poor image quality or images found to be corrupted during processing, affecting further analysis;(5) missing key data required for GNRI calculation, MoCA assessment, or WMH quantification.

Baseline clinical data were collected retrospectively after admission, covering age, sex, education level, body mass index (BMI), histories of hypertension, coronary heart disease, diabetes, hyperlipidemia, and stroke, along with smoking and alcohol use. Laboratory measurements from fasting blood samples included homocysteine (HCY), vitamin B12, folic acid, albumin, glycated hemoglobin (HbA1c), fasting glucose, triglycerides (TG), total cholesterol (TC), low-density lipoprotein (LDL), very-low-density lipoprotein (VLDL), and high-density lipoprotein (HDL).

### 2.2 Nutritional assessment

The GNRI was used to assess the nutritional status of patients, calculated with the formula: [1.489 × serum albumin (g/L) + 41.7 × current weight (kg)/ideal body weight (kg)] (20). If the current body weight is greater than the ideal weight, the weight-to-ideal weight ratio is set to 1. The ideal body weight was determined using the Lorenz formula based on the patient’s height and sex (21).

### 2.3 Cognitive function test

Cognitive function was assessed using the MoCA. The MoCA assessment covers eight cognitive domains, including visuospatial and executive functions, naming, attention, memory, delayed recall, language, abstract thinking, and orientation. The total score is 30 points. For participants with ≤12 years of education, one point was added to the MoCA score. An education-adjusted score below 26 indicated cognitive impairment, while a score of 26 or above indicated normal cognitive function.

### 2.4 Quantification of WMH volume

**Figure 1:**
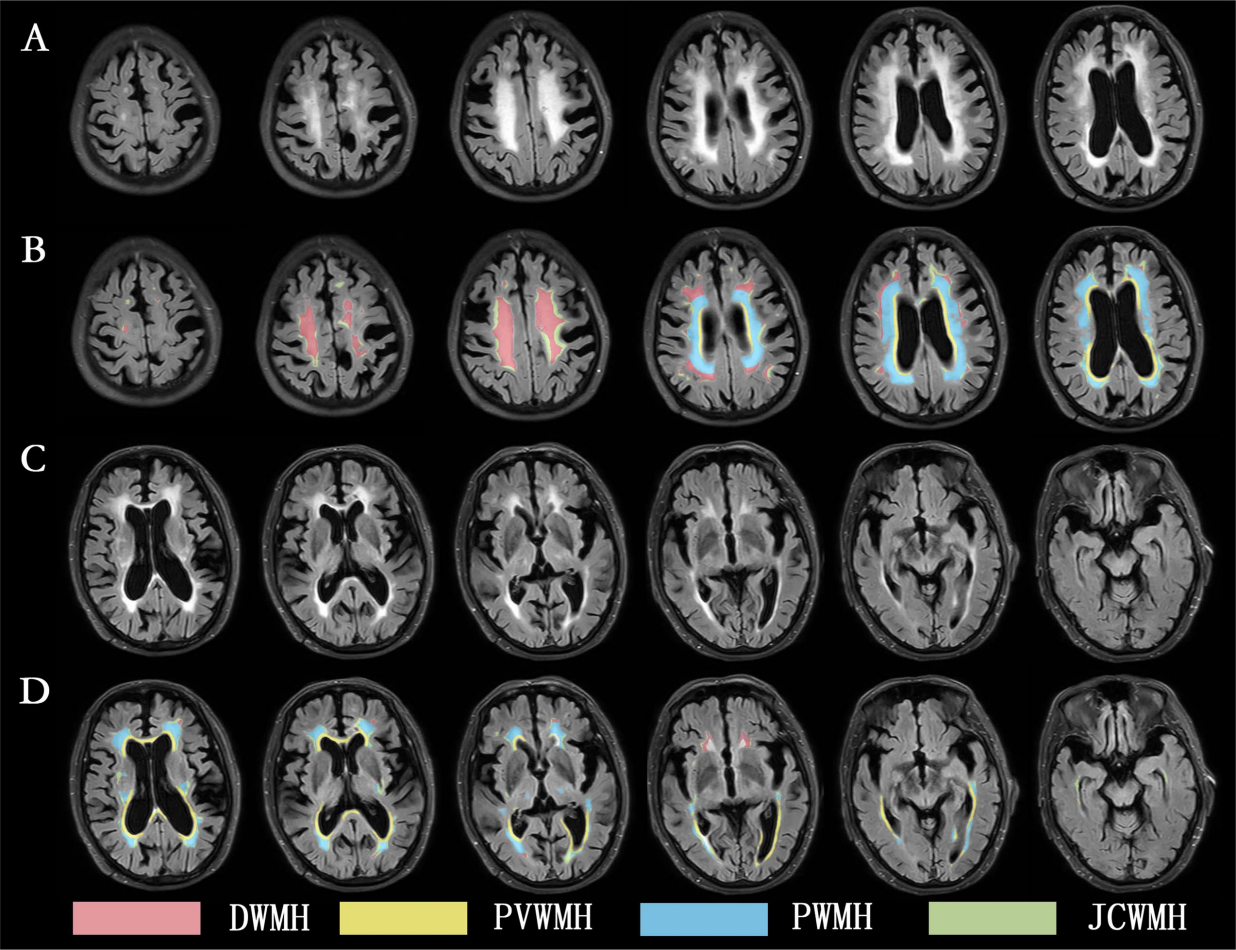
T2-FLAIR image and corresponding processed image. **(A,C)** T2-FLAIR image; **(B,D)** corresponding processed image.

In line with our established methodology, T2-FLAIR images were processed using the United Imaging’s Intelligent Analysis System for Cerebral Small Vessel Disease (22). This system performs segmentation of WMH through a convolutional neural network based on a two-dimensional VB-Net architecture (23). From the segmentation results, the following volumetric parameters were computed: total WMH volume (defined as the sum of periventricular, deep, and juxtacortical WMH volumes), periventricular WMH volume (PVWMH, comprising WMH along the lateral ventricular rim and paraventricular regions), deep WMH volume (DWMH), and juxtacortical WMH volume (JCWMH). A schematic representation of these volumetric classifications is provided in Figure 1.

### 2.5 Assessment of total CSVD burden

For subgroup analyses, the total CSVD burden score was evaluated using conventional MRI markers, including lacunes, cerebral microbleeds, basal ganglia enlarged perivascular spaces, and WMH severity. Each marker was scored as 1 point according to established criteria, with a total score ranging from 0 to 4. Patients were further divided into a low-burden group (score 0–2) and a high-burden group (score 3–4).

### 2.6 Statistical analysis

Data were analyzed using SPSS Statistics 25 and visualized using GraphPad Prism 10. The normality of continuous variables was assessed before analysis. Continuous variables conforming to a normal distribution are expressed as mean ± standard deviation, and those with a skewed distribution are expressed as median and interquartile range (IQR). Categorical variables are summarized as frequency counts and percentages. For between-group comparisons, continuous variables were analyzed using the t-test or Mann–Whitney U test, and categorical variables were analyzed using the *χ*^2^ test. Spearman correlation analysis was used to evaluate the associations among GNRI, WMH volume, and MoCA scores. WMH volumes were natural log-transformed using ln(volume + 1) before linear regression and mediation analyses. Subsequently, multivariable linear regression analyses were conducted to examine the associations of GNRI and log-transformed WMH volumes with MoCA scores after adjusting for age, sex, education level, hypertension, diabetes mellitus, previous stroke events, coronary heart disease, hyperlipidemia, smoking, drinking, and VLDL. Mediation analysis was carried out using the SPSS PROCESS Macro v3.4 (Model 4) to examine whether log-transformed WMH volume mediated the association between GNRI and MoCA scores after adjusting for the same covariates (44). Indirect effects were estimated using 5,000 bootstrap samples. Complete-case analysis was used for the adjusted regression and mediation models. Sensitivity mediation analyses were performed by excluding VLDL from the covariates, and subgroup mediation analyses were conducted according to age, sex, and CSVD burden. A two-tailed *p*-value < 0.05 was considered statistically significant.

## 3 Results

### 3.1 Baseline data

**Figure 2:**
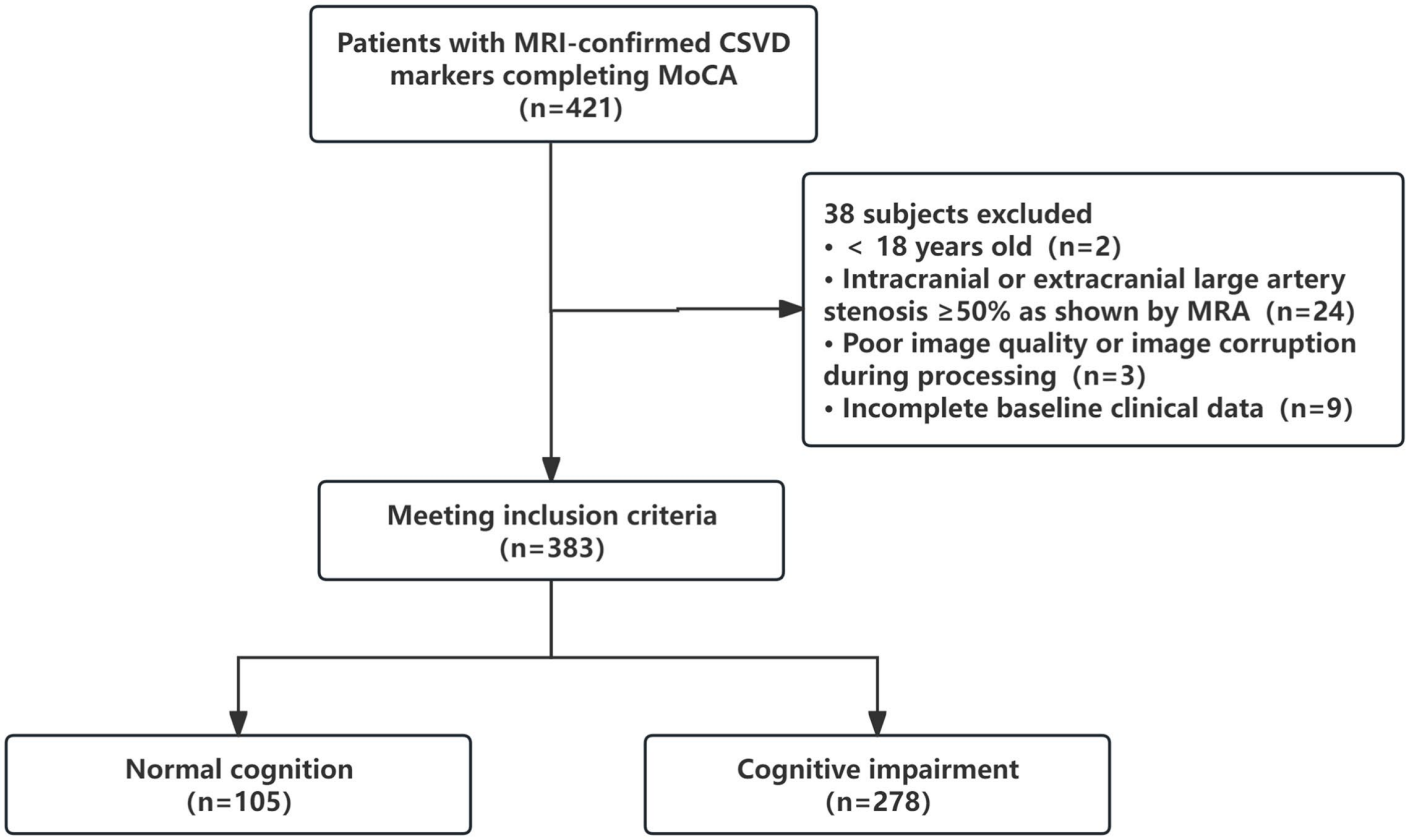
Flowchart for the selection of patients.

A total of 421 patients were initially screened, of whom 383 met the inclusion and exclusion criteria and were enrolled in the study (Figure 2). The mean age of the participants was 67.02 ± 10.73 years, and 227 (59.30%) were male. Based on MoCA scores, 105 patients (28.77%) were classified as cognitively normal and 278 (76.16%) as having cognitive impairment. The median MoCA score was 22.0 (19.0, 26.0).

### 3.2 Comparison of clinical characteristics and WMH volume between the two groups of patients

**Figure 3:**
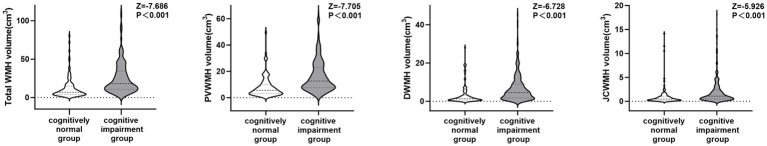
Comparison of WMH volumes between the two groups.

The cognitive impairment group demonstrated significantly lower GNRI and MoCA scores compared to the cognitively normal group (*p* < 0.05). In addition, the cognitive impairment group showed a lower prevalence of prior hyperlipidemia, reduced VLDL levels, and higher age relative to the cognitively normal group (*p* < 0.05, Table 1). Furthermore, significantly larger volumes of Total WMH, PVWMH, DWMH, and JCWMH were observed in the cognitive impairment group compared to the cognitively normal group (*p* < 0.05, Figure 3).

**Table 1:** Comparison of clinical characteristics between the two groups.

Variables	Overall (*N* = 383)	Cognitively normal group (*N* = 105)	Cognitive impairment group (N = 278)	*χ*^2^/*t*/*Z*	*P*
Age (years), mean ± SD	67.02 ± 10.73	62.07 ± 11.43	69.06 ± 10.03	−4.252	*P* < 0.001
Sex male, *n* (%)	227 (59.30)	62 (59.00)	165 (59.40)	0.003	0.957
BMI	24.46 (22.58, 26.66)	24.93 (23.13, 26.28)	24.22 (22.04, 26.71)	−1.480	0.139
Education level ≤12 years, *n* (%)	158 (41.30)	36 (34.30)	122 (43.90)	2.898	0.089
Past medical history					
Hypertension, *n* (%)	265 (69.2)	72 (68.6)	193 (69.4)	0.026	0.872
Diabetes, *n* (%)	137 (35.8)	41 (39.0)	96 (34.5)	0.676	0.411
Previous stroke events, *n* (%)	248 (64.8)	63 (60.0)	185 (66.5)	1.431	0.232
Hyperlipidemia, *n* (%)	136 (35.5)	47 (44.8)	89 (32.0)	5.408	0.020
Coronary heart disease, *n* (%)	67 (17.5)	21 (20.0)	46 (16.5)	0.630	0.427
Smoking, *n* (%)	103 (27.1)	28 (26.7)	75 (27.3)	0.014	0.905
Drinking, *n* (%)	79 (20.8)	25 (23.8)	54 (19.6)	0.804	0.370
Blood test results					
Hcy, median [IQR], μmol/L	13.84 (11.24, 17.98)	13.73 (10.90, 18.38)	13.90 (11.30, 18.11)	−0.374	0.708
VB12 median [IQR], pg./ml	356.4 (225.80, 614.50)	404.00 (243.60, 643.65)	323.00 (215.58, 599.93)	−1.321	0.186
Folic acid, median [IQR], ng/ml	9.12 (6.15, 13.4)	8.92 (5.82, 13.18)	8.45 (6.18, 12.53)	−0.730	0.465
Albumin, median [IQR],g/L	41.74 (39.50, 44.00)	43.20 (41.56, 45.28)	40.95 (39.08, 43.25)	−6.146	*P* < 0.001
HbA1C,median [IQR], %	5.90 (5.60, 6.65)	6.00 (5.60, 6.50)	5.85 (5.50, 6.70)	−0.203	0.839
FBG, median [IQR], mmol/L	5.64 (4.99, 7.07)	5.42 (4.87, 7.23)	5.56 (4.92, 6.80)	−1.013	0.311
TC, median [IQR], mmol/L	4.06 (3.40, 4.90)	4.13 (3.58, 4.93)	4.07 (3.44, 4.76)	−1.117	0.264
TG, median [IQR], mmol/L	1.06 (0.75, 1.60)	1.16 (0.80, 1.62)	1.02 (0.70, 1.60)	−1.285	0.199
HDL, median [IQR], mmol/L	1.12 (1.00, 1.35)	1.12 (0.97, 1.44)	1.13 (1.00, 1.31)	−0.234	0.815
LDL, median [IQR], mmol/L	2.47 (1.77, 3.11)	2.50 (1.93, 3.27)	2.49 (1.91, 3.09)	−0.147	0.883
VLDL, median [IQR], mmol/L	0.54 (0.37, 0.78)	0.59 (0.40, 0.87)	0.52 (0.34, 0.77)	−2.119	0.034
GNRI	103.34 (99.47, 106.62)	105.73 (103.58, 108.81)	102.00 (98.36, 105.28)	−6.799	*P* < 0.001
MOCA, median [IQR], point	22.0 (19.0, 26.0)	27.0 (26.0, 28.0)	20.0 (17.0, 22.0)	−15.140	*P* < 0.001

### 3.3 Associations of GNRI, WMH, and cognitive function

The relationship between GNRI, WMH volume, and cognitive function was examined using Spearman correlation analysis. As shown in Table 2, cognitive function was positively correlated with GNRI (*r* = 0.454, *p* < 0.001). Furthermore, all WMH volumes demonstrated significant negative correlations with cognitive function: Total WMH (*r* = −0.538, *p* < 0.001), PVWMH (*r* = −0.544, *p* < 0.001), DWMH (*r* = −0.469, *p* < 0.001), and JCWMH (*r* = −0.430, *p* < 0.001). Additionally, GNRI showed significant negative correlations with all WMH volumes: Total WMH (*r* = −0.482, *p* < 0.001), PVWMH (*r* = −0.493, *p* < 0.001), DWMH (*r* = −0.403, *p* < 0.001), and JCWMH (*r* = −0.423, *p* < 0.001).

**Table 2:** Correlation analysis of GNRI, WMH volumes, and cognitive function.

Interactions between variables		Correlation coefficient (r)	*p*-value
GNRI-MoCA		0.454	*P* < 0.001
GNRI-WMH			*P* < 0.001
GNRI	Total WMH	−0.482	*P* < 0.001
GNRI	PVWMH	−0.493	*P* < 0.001
GNRI	DWMH	−0.403	*P* < 0.001
GNRI	JCWMH	−0.423	*P* < 0.001
WMH-MoCA			*P* < 0.001
Total WMH	MoCA	−0.538	*P* < 0.001
PVWMH	MoCA	−0.544	*P* < 0.001
DWMH	MoCA	−0.469	*P* < 0.001
JCWMH	MoCA	−0.430	*P* < 0.001

### 3.4 Multiple regression models of GNRI, log-transformed WMH volumes, and MoCA scores

Multivariable linear regression models were adjusted for age, sex, education level, hypertension, diabetes mellitus, previous stroke events, coronary heart disease, hyperlipidemia, smoking, drinking, and VLDL. Higher GNRI scores were significantly associated with lower log-transformed total WMH, PVWMH, DWMH, and JCWMH volumes (all *p* < 0.001). Higher GNRI scores were also significantly associated with higher MoCA scores (*p* < 0.001). In separate models, greater log-transformed total WMH, PVWMH, DWMH, and JCWMH volumes were significantly associated with lower MoCA scores (all *p* < 0.001). When GNRI and each log-transformed WMH volume were simultaneously included in the models, the association between GNRI and MoCA scores was attenuated, particularly in the models including total WMH and PVWMH. Log-transformed WMH volumes remained significantly associated with MoCA scores in all models, as detailed in Table 3.

**Table 3:** Multivariable regression analysis among GNRI, log-transformed WMH volumes, and MoCA scores.

Outcome variable	Predictor variable	*R*	*R* ^2^	*F*	*B*	95% CI	*P*
Total WMH							
ln-total WMH	GNRI	0.507	0.257	9.644	−0.061	(−0.076, −0.046)	<0.001
MoCA	GNRI	0.428	0.183	6.236	0.211	(0.126, 0.296)	<0.001
MoCA	ln-total WMH	0.573	0.329	13.626	−2.591	(−3.098, −2.084)	<0.001
MoCA	GNRI	0.577	0.333	12.779	0.062	(−0.022, 0.146)	0.150
	ln-total WMH				−2.428	(−2.981, −1.876)	<0.001
PVWMH							
ln-PVWMH	GNRI	0.531	0.282	10.934	−0.055	(−0.069, −0.042)	<0.001
MoCA	GNRI	0.428	0.183	6.236	0.211	(0.126, 0.296)	<0.001
MoCA	ln-PVWMH	0.577	0.333	13.888	−2.942	(−3.509, −2.374)	<0.001
MoCA	GNRI	0.580	0.337	12.991	0.058	(−0.026, 0.142)	0.178
	ln-PVWMH				−2.769	(−3.389, −2.148)	<0.001
DWMH							
ln-DWMH	GNRI	0.402	0.162	5.380	−0.055	(−0.072, −0.039)	<0.001
MoCA	GNRI	0.428	0.183	6.236	0.211	(0.126, 0.296)	<0.001
MoCA	ln-DWMH	0.526	0.277	10.661	−2.090	(−2.581, −1.599)	<0.001
MoCA	GNRI	0.539	0.29	10.474	0.107	(0.023, 0.192)	0.013
	ln-DWMH				−1.867	(−2.385, −1.349)	<0.001
JCWMH							
ln-JCWMH	GNRI	0.423	0.179	6.048	−0.04	(−0.052, −0.029)	<0.001
MoCA	GNRI	0.428	0.183	6.236	0.211	(0.126, 0.296)	<0.001
MoCA	ln-JCWMH	0.511	0.261	9.833	−2.83	(−3.540, −2.119)	<0.001
MoCA	GNRI	0.524	0.275	9.718	0.111	(0.025, 0.197)	0.012
	ln-JCWMH				−2.488	(−3.241, −1.735)	<0.001

### 3.5 Log-transformed WMH volume as a mediator between GNRI and cognitive function

**Figure 4:**
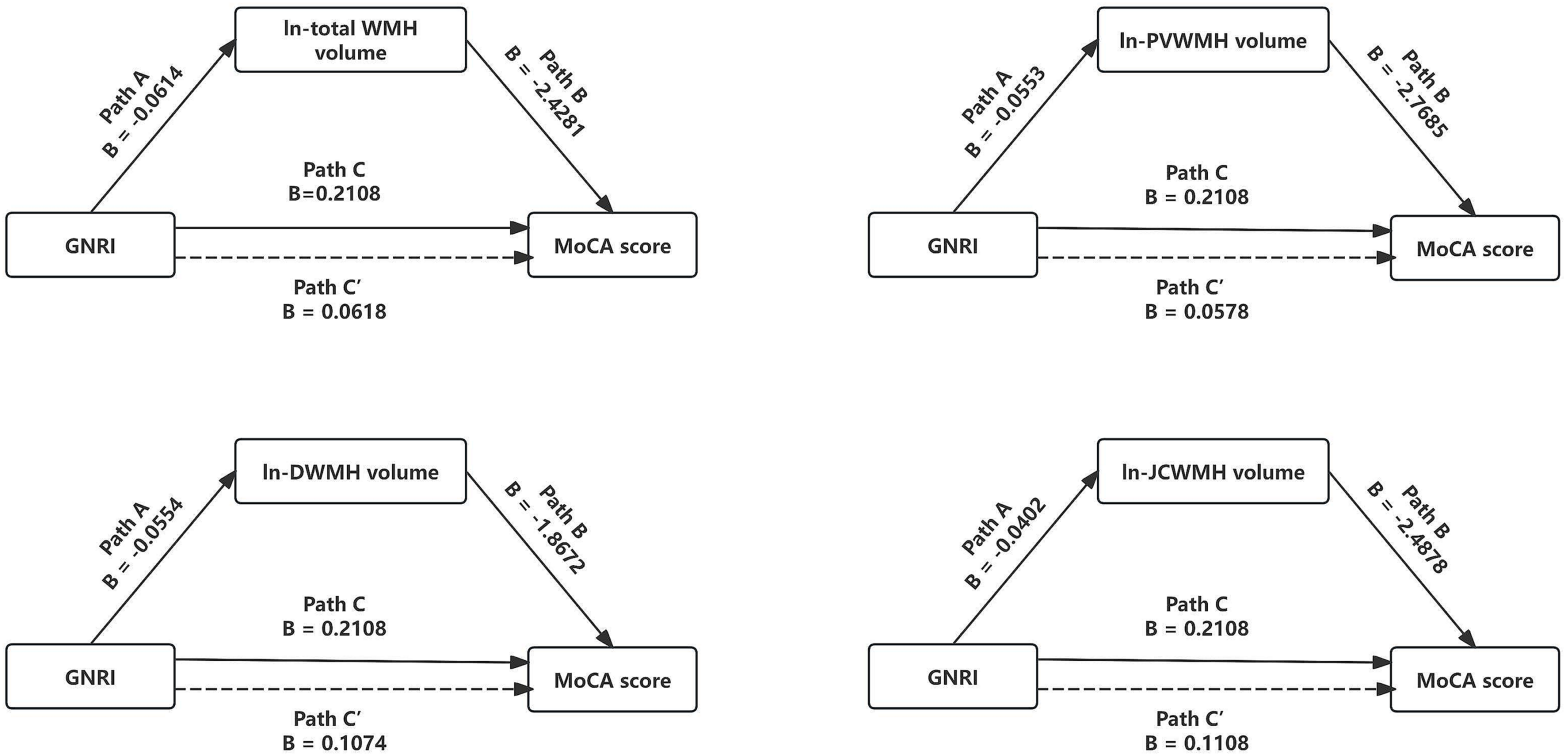
Mediation model diagram. The mediation model illustrates the mediating role of log-transformed WMH volumes in the association between GNRI and MoCA scores. Results were adjusted for age, sex, education level, hypertension, diabetes mellitus, previous stroke events, coronary heart disease, hyperlipidemia, smoking, drinking, and VLDL. Path A represents the association between GNRI and log-transformed WMH volumes; Path B represents the association between log-transformed WMH volumes and MoCA scores; Path C represents the total association between GNRI and MoCA scores; and Path C′ represents the direct association between GNRI and MoCA scores. Path coefficients are presented as unstandardized regression coefficients.

Mediation analysis showed that log-transformed total WMH significantly mediated the association between GNRI and MoCA scores, with a relative mediation effect of 70.7% (indirect effect = 0.1490, 95% bootstrap CI: 0.1007, 0.2054). Further analysis of individual WMH subtypes showed that log-transformed PVWMH, DWMH, and JCWMH also significantly mediated the association between GNRI and MoCA scores. Among these subtypes, PVWMH showed the largest relative mediation effect, accounting for 72.6%, followed by DWMH and JCWMH, which accounted for 49.1 and 47.5%, respectively, as shown in Table 4. The data-driven mediation model is illustrated in Figure 4.

**Table 4:** The mediating effect of log-transformed WMH volumes between GNRI and MoCA scores.

Effect type	Effect value	SE/BootSE	95% CI	Relative mediation effect
ln-total WMH				
Total effect	0.2108	0.0433	(0.1256, 0.2961)	1
Direct effect (GNRI → MoCA)	0.0618	0.0429	(−0.0225, 0.1461)	0.293
Indirect effect (GNRI → ln-total WMH → MoCA)	0.1490	0.0267	(0.1007, 0.2054)	0.707
ln-PVWMH				
Total effect	0.2108	0.0433	(0.1256, 0.2961)	1
Direct effect (GNRI → MoCA)	0.0578	0.0428	(−0.0265, 0.1420)	0.274
Indirect effect (GNRI → ln-PVWMH → MoCA)	0.1531	0.0266	(0.1049, 0.2100)	0.726
ln-DWMH				
Total effect	0.2108	0.0433	(0.1256, 0.2961)	1
Direct effect (GNRI → MoCA)	0.1074	0.0430	(0.0227, 0.1920)	0.510
Indirect effect (GNRI → ln-DWMH → MoCA)	0.1035	0.0233	(0.0619, 0.1529)	0.491
ln-JCWMH				
Total effect	0.2108	0.0433	(0.1256, 0.2961)	1
Direct effect (GNRI → MoCA)	0.1108	0.0437	(0.0248, 0.1967)	0.526
Indirect effect (GNRI → ln-JCWMH → MoCA)	0.1001	0.0227	(0.0601, 0.1484)	0.475

### 3.6 Sensitivity and subgroup mediation analyses

Sensitivity mediation analyses excluding VLDL from the covariates showed that the indirect effects of log-transformed total WMH, PVWMH, DWMH, and JCWMH remained significant. Subgroup mediation analyses stratified by age, sex, and CSVD burden also showed significant indirect effects across all subgroups. The detailed results are presented in Supplementary Tables 1, 2.

## 4 Discussion

This retrospective cross-sectional study investigated the associations among nutritional status, WMH, and cognitive function by assessing nutritional status using the GNRI, quantitatively analyzing the volumes of various WMH subtypes on MRI images with AI-based software, and evaluating cognition with the MoCA. The results demonstrated that both malnutrition and increased WMH volume were associated with cognitive impairment. This study further showed that log-transformed WMH volume mediated the association between GNRI and cognitive impairment in the mediation model. These findings suggest that WMH may be a quantifiable imaging marker involved in the association between nutritional status and cognitive function, providing a potential perspective for early identification and risk assessment.

In terms of nutritional status, the significant findings regarding GNRI scores further highlight the association between poor nutritional health and cognitive performance. This study demonstrated that the MoCA score of CSVD patients was positively correlated with GNRI. Regression analysis indicated that lower GNRI was associated with cognitive impairment. Two cross-sectional studies showed that patients with malnutrition, as defined by the GNRI, had an increased incidence of cognitive decline compared to patients with normal nutritional status, and lower GNRI values were associated with functional decline in multiple cognitive domains (10, 24). A prospective longitudinal study in China also reported a significant relationship between the GNRI and cognition among older adults, with subjects experiencing more severe malnutrition demonstrating worse cognitive performance (9). Consistent with prior evidence, this study supports the link between decreased GNRI and cognitive impairment in patients with CSVD.

The GNRI is an objective indicator calculated from height, body weight, and serum albumin level to assess nutritional status. Therefore, the association between reduced GNRI and cognitive impairment may be partly related to albumin and BMI. Our results indicated that lower albumin levels were correlated with cognitive impairment. A study involving elderly Korean veterans and their family members found an inverse association between serum albumin levels and cognitive function scores (25). Increased albumin levels are associated with a reduced incidence of cognitive dysfunction (26). Albumin serves as a critical marker of nutritional status (27), and its association with cognitive impairment may be related to oxidative stress and compromised microcirculation (26). BMI is a fundamental and commonly employed measure for evaluating nutritional status in individuals and populations (28). Studies have shown a significant correlation between BMI and cognitive function in elderly populations (29). Individuals with a higher BMI have a lower risk of severe cognitive impairment and exhibit a slower rate of cognitive decline (30). However, no significant correlation between BMI and cognitive function was observed in this study. Since BMI cannot differentiate body composition—specifically, it does not distinguish between fat mass and lean body mass (such as muscle and bone)—it may inaccurately assess true nutritional status (31). In contrast, the GNRI integrates both albumin and body weight, making it a more representative indicator of nutritional function (8). Therefore, our findings support an association between lower GNRI and cognitive impairment in patients with CSVD.

Regarding the relationship between malnutrition and the volume of WMH, the findings indicate a positive correlation between malnutrition and WMH. Multiple studies have reported that malnutrition is independently associated with WMH burden. A study of an elderly population showed that individuals at risk of malnutrition had approximately a 93% higher likelihood of having severe WMH compared to those with good nutritional status, while the likelihood was 180% higher in those with confirmed malnutrition (32). Another study focusing on patients with CSVD reported that higher nutritional risk scores were independently associated with greater overall CSVD burden and its imaging markers, including WMH (18). The association is also significant in specific patient populations: among peritoneal dialysis patients, indicators reflecting malnutrition-inflammation status were significantly linked to CSVD, including WMH (33). Furthermore, the association between low serum albumin and WMH was particularly prominent in populations with alcohol use disorder and HIV infection (34).

Investigating WMH and their association with cognitive function provides an important perspective for understanding cognitive decline. The presence of WMH is primarily linked to microvascular ischemia and perivascular injury resulting from cerebrovascular risk factors, which contribute to reduced cerebral blood flow and tissue damage (35–37). The pathophysiological basis indicates that WMH contributes to cognitive impairment via mechanisms including ischemia and the disruption of neural pathways essential for cognitive processing (38). Total WMH volume correlated inversely with cognitive performance in our study, which aligns with previous studies identifying WMH as a marker of vascular injury and neurodegeneration (39, 40). Importantly, mediation analysis showed that log-transformed WMH volume mediated the association between GNRI and MoCA scores in patients with CSVD. Potential biological explanations may involve the following aspects: (1) The GNRI is essentially an albumin-based index adjusted for height and weight. Serum albumin is a major component responsible for maintaining plasma colloid osmotic pressure and blood volume (26). A decrease in this index may reflect poorer nutritional status and reduced albumin levels, which may be associated with impaired central nervous system perfusion and compromised microvascular perfusion. Such perfusion abnormalities may be associated with the development of WMH and cognitive decline. (2) Oxidative stress plays a key role in the pathophysiology of cognitive impairment, a process in which nutrition and inflammation interact (41). Serum albumin plays a crucial antioxidant role *in vivo* through its sulfhydryl groups, polysulfide chains, and binding capacity (42). Reduced albumin levels may weaken the body’s capacity to counteract oxidative stress, which may be associated with WMH formation and cognitive decline (43). Therefore, WMH may help explain the association between malnutrition and cognitive decline, possibly in relation to microvascular perfusion and oxidative stress.

This study has several limitations. First, the retrospective single-center design may introduce selection bias and limit the generalizability of the findings. Larger multicenter studies are needed to further validate the associations among nutritional status, WMH burden, and cognitive function in patients with CSVD. Second, because of the cross-sectional design, the temporal and causal relationships among GNRI, WMH burden, and cognitive function could not be determined. Therefore, the observed mediation effect should be interpreted as a statistical association rather than evidence of a causal pathway. Reverse causality, such as cognitive impairment leading to poor nutritional status, also cannot be excluded. Third, although multiple potential confounders were adjusted for, residual confounding cannot be completely excluded. Information on physical activity, detailed lifestyle factors, and pre-admission medication use, such as statins and antiplatelet agents, was not available. Fourth, although the GNRI is an objective and convenient index for assessing nutritional risk, it mainly reflects serum albumin and body weight status and cannot fully capture dietary patterns, micronutrient deficiencies, sarcopenia, body composition, or inflammation-related nutritional abnormalities. Finally, MoCA is a screening tool and may not fully characterize domain-specific cognitive impairment; therefore, comprehensive neuropsychological assessments should be considered in future studies.

## 5 Conclusion

In summary, this study demonstrates that in patients with CSVD, cognitive function is associated with WMH and nutritional status. Furthermore, it shows that log-transformed WMH volume mediated the association between GNRI and cognitive impairment in the mediation model. By identifying WMH as a potential link between nutritional status and cognitive function, this research provides a visualizable perspective for understanding cognitive impairment in patients with CSVD. Future studies should employ longitudinal designs and comprehensive nutritional assessments to further clarify these associations and evaluate whether WMH volume can serve as a quantitative and visualizable measure in studies of nutritional interventions and cognitive outcomes in CSVD.

## Data Availability

The raw data supporting the conclusions of this article will be made available by the authors, without undue reservation.
