# Supplementary material for: The mediating role of white matter hyperintensity in the association between malnutrition and cognitive impairment in patients with cerebral small vessel disease
Source: Front Neurol. 2026 Jul 27;17:1841396. doi: 10.3389/fneur.2026.1841396 (PMC13454999; doi:10.3389/fneur.2026.1841396)
Supplement: Supplementary file 1 [file Table_1.DOC]

**Supplementary Table 1. Sensitivity mediation analysis excluding VLDL from the covariates**

| **Mediator** | **Effect type** | **Effect value** | **SE/BootSE** | **95% CI** | **Relative mediation effect** |
| --- | --- | --- | --- | --- | --- |
| **ln-total WMH** | Total effect | 0.2393 | 0.04 | (0.1606, 0.3180) | 1 |
|  | Direct effect (GNRI→ MoCA) | 0.0853 | 0.0401 | (0.0065, 0.1641) | 0.356 |
|  | Indirect effect (GNRI→ ln-total WMH→ MoCA) | 0.1540 | 0.0254 | (0.1081, 0.2078) | 0.644 |
| **ln-PVWMH** | Total effect | 0.2393 | 0.04 | (0.1606, 0.3180) | 1 |
|  | Direct effect (GNRI→ MoCA) | 0.0815 | 0.04 | (0.0027, 0.1602) | 0.341 |
|  | Indirect effect (GNRI→ ln-PVWMH→ MoCA) | 0.1578 | 0.0254 | (0.1119, 0.2097) | 0.659 |
| **ln-DWMH** | Total effect | 0.2393 | 0.04 | (0.1606, 0.3180) | 1 |
|  | Direct effect (GNRI→ MoCA) | 0.1309 | 0.04 | (0.0522, 0.2096) | 0.547 |
|  | Indirect effect (GNRI→ ln-DWMH→ MoCA) | 0.1084 | 0.0224 | (0.0686, 0.1569) | 0.453 |
| **ln-JCWMH** | Total effect | 0.2393 | 0.04 | (0.1606, 0.3180) | 1 |
|  | Direct effect (GNRI→ MoCA) | 0.1413 | 0.0409 | (0.0609, 0.2217) | 0.590 |
|  | Indirect effect (GNRI→ ln-JCWMH→ MoCA) | 0.0980 | 0.0221 | (0.0581, 0.1440) | 0.410 |

Note: Sensitivity mediation analyses were performed using PROCESS macro Model 4 after excluding VLDL from the covariates. GNRI was entered as the independent variable, MoCA score as the dependent variable, and log-transformed WMH volume [ln(volume + 1)] as the mediator. Models were adjusted for age, sex, education level, hypertension, diabetes mellitus, previous stroke events, coronary artery disease, hyperlipidemia, smoking, and drinking. Indirect effects were estimated using 5,000 bootstrap samples, and 95% CIs are presented as (lower limit, upper limit). GNRI, Geriatric Nutritional Risk Index; WMH, white matter hyperintensity; PVWMH, periventricular WMH; DWMH, deep WMH; JCWMH, juxtacortical WMH; MoCA, Montreal Cognitive Assessment; VLDL, very-low-density lipoprotein.

**Supplementary Table 2. Subgroup mediation analysis stratified by age, sex, and CSVD burden**

| **Subgroup variable** | **Subgroup** | **Mediator** | **N** | **Indirect effect** | **BootSE** | **95% CI** |
| --- | --- | --- | --- | --- | --- | --- |
| Age | <65 years | ln-total WMH | 132 | 0.2201 | 0.0584 | (0.1232, 0.3485) |
| Age | <65 years | ln-PVWMH | 132 | 0.2302 | 0.0609 | (0.1265, 0.3661) |
| Age | <65 years | ln-DWMH | 132 | 0.1697 | 0.0543 | (0.0776, 0.2885) |
| Age | <65 years | ln-JCWMH | 132 | 0.1605 | 0.0593 | (0.0651, 0.2968) |
| Age | ≥65 years | ln-total WMH | 215 | 0.1167 | 0.0266 | (0.0693, 0.1739) |
| Age | ≥65 years | ln-PVWMH | 215 | 0.1227 | 0.0258 | (0.0757, 0.1759) |
| Age | ≥65 years | ln-DWMH | 215 | 0.0702 | 0.0226 | (0.0319, 0.1186) |
| Age | ≥65 years | ln-JCWMH | 215 | 0.0785 | 0.0218 | (0.0401, 0.1259) |
| Sex | Male | ln-total WMH | 201 | 0.1489 | 0.0352 | (0.0900, 0.2273) |
| Sex | Male | ln-PVWMH | 201 | 0.1505 | 0.0351 | (0.0885, 0.2251) |
| Sex | Male | ln-DWMH | 201 | 0.1057 | 0.0329 | (0.0509, 0.1782) |
| Sex | Male | ln-JCWMH | 201 | 0.0938 | 0.0284 | (0.0450, 0.1586) |
| Sex | Female | ln-total WMH | 146 | 0.1528 | 0.0443 | (0.0754, 0.2485) |
| Sex | Female | ln-PVWMH | 146 | 0.1599 | 0.0437 | (0.0825, 0.2521) |
| Sex | Female | ln-DWMH | 146 | 0.1013 | 0.0393 | (0.0359, 0.1886) |
| Sex | Female | ln-JCWMH | 146 | 0.1123 | 0.038 | (0.0449, 0.1942) |
| CSVD burden | Low burden, score 0–2 | ln-total WMH | 197 | 0.0741 | 0.0216 | (0.0369, 0.1218) |
| CSVD burden | Low burden, score 0–2 | ln-PVWMH | 197 | 0.0742 | 0.022 | (0.0375, 0.1221) |
| CSVD burden | Low burden, score 0–2 | ln-DWMH | 197 | 0.0412 | 0.018 | (0.0121, 0.0816) |
| CSVD burden | Low burden, score 0–2 | ln-JCWMH | 197 | 0.0358 | 0.0157 | (0.0090, 0.0705) |
| CSVD burden | High burden, score 3–4 | ln-total WMH | 150 | 0.1209 | 0.0409 | (0.0464, 0.2067) |
| CSVD burden | High burden, score 3–4 | ln-PVWMH | 150 | 0.1283 | 0.0435 | (0.0489, 0.2213) |
| CSVD burden | High burden, score 3–4 | ln-DWMH | 150 | 0.0704 | 0.0298 | (0.0175, 0.1348) |
| CSVD burden | High burden, score 3–4 | ln-JCWMH | 150 | 0.0751 | 0.0337 | (0.0182, 0.1502) |

Note: Subgroup mediation analyses were performed using PROCESS macro Model 4. GNRI was entered as the independent variable, MoCA score as the dependent variable, and log-transformed WMH volume [ln(volume + 1)] as the mediator. Age-stratified models were adjusted for sex, smoking, drinking, hypertension, diabetes mellitus, previous stroke events, hyperlipidemia, coronary artery disease, education level, and VLDL. Sex-stratified models were adjusted for age, smoking, drinking, hypertension, diabetes mellitus, previous stroke events, hyperlipidemia, coronary artery disease, education level, and VLDL. CSVD burden-stratified models were adjusted for age, sex, smoking, drinking, hypertension, diabetes mellitus, previous stroke events, hyperlipidemia, coronary artery disease, education level, and VLDL. Indirect effects were estimated using 5,000 bootstrap samples, and 95% CIs are presented as (lower limit, upper limit). Complete-case analysis was used for subgroup mediation analyses. GNRI, Geriatric Nutritional Risk Index; MoCA, Montreal Cognitive Assessment; CSVD, cerebral small vessel disease; WMH, white matter hyperintensity; PVWMH, periventricular WMH; DWMH, deep WMH; JCWMH, juxtacortical WMH; VLDL, very-low-density lipoprotein.
